# Clinical Significance of Serum NEDD9 Levels in Patients with Pancreatic Cancer

**DOI:** 10.3390/biom8040169

**Published:** 2018-12-10

**Authors:** Cigdem Usul Afsar, Mehmet Karabulut, Senem Karabulut, Safiye Tokgoz Ozal, Murat Cikot, Murat Serilmez, Faruk Tas

**Affiliations:** 1 Acıbadem Bakırkoy Hospital, Department of Internal Medicine and Medical Oncology, Medical Faculty, Acıbadem Mehmet Ali Aydınlar University, Zeytinlik Mah. Halit Ziya Usaklıgil Cad. No: 1, Bakırkoy, 34140 Istanbul, Turkey; 2 Department of General Surgery, Bakırkoy Dr Sadi Konuk Education and Research Hospital, Health Sciences University, 34147 Istanbul, Turkey; mehmetkarabulut@hotmail.com (M.K.); mehmet_mac@hotmail.com (M.C.); 3 Department of Medical Oncology, Institute of Oncology, Istanbul University, 34452 Fatih/Istanbul, Turkey; drsenemkarabulut@gmail.com (S.K.); ftas@yahoo.com (F.T.); 4 Department of Radiology, Bakırkoy Dr Sadi Konuk Education and Research Hospital, Health Sciences University, 34147 Istanbul, Turkey; safiyeozal@gmail.com; 5 Department of Basic Oncology, Institute of Oncology, Istanbul University, 34452 Istanbul, Turkey; serilmez55@hotmail.com

**Keywords:** NEDD9, pancreatic cancer, serum, diagnostic

## Abstract

Introduction: Pancreatic cancer (PC) is a lethal malignancy. Various diagnostic, predictive, and prognostic biomarkers have been evaluated. This study was conducted to investigate the serum levels of neural precursor cell expressed developmentally downregulated protein 9 (NEDD9) in patients with PC and the relationship between tumor progression and known prognostic parameters. Materials and Methods: Serum samples were obtained on first admission before any treatment. Serum NEDD9 levels were determined using enzyme-linked immunosorbent assay (ELISA). Age- and sex-matched healthy controls were included in the analysis. Results: In a three year period, 32 patients with a pathologically-confirmed diagnosis of PC were enrolled in this study. The median age at diagnosis was 61 years, range 38 to 84 years; the majority of the patients in the group were men (*n* = 20, 62.5%). The tumor was located in the head of pancreas in 21 (65.6%) patients. Forty-one percent of 17 metastatic patients who received palliative CTx (chemotherapy) were CTx-responsive. The baseline serum NEDD9 levels were significantly higher in patients with PA than in the control group (*p* = 0.03). Median OS of the whole group were 27 ± 7.3 weeks. Alcohol intake, performance status, and LDH levels were found to be significant prognostic factors (*p* = 0.006, *p* < 0.001, and *p* < 0.001, respectively). However, serum NEDD9 levels had no significantly effect on progression free survival (PFS) and overall survival (OS) (*p* = 0.71 and *p* = 0.58, respectively). Conclusions: NEDD9 is identified as a secretory biomarker for PC but it has no prognostic role.

## 1. Introduction

Pancreatic cancer (PC) is one of the most fatal cancers with an extremely poor prognosis even in its early stages. It is found to be metastatic at presentation in approximately 50% of cases [1,2]. It is the fourth leading cause of cancer-related death in the United States, and is second only to colorectal cancer as a cause of digestive cancer-related death [2]. Carcinoma of the exocrine pancreas is a genetic disease that is caused by inherited and acquired mutations in specific cancer-associated genes [3,4]. The patterns of genetic alterations identified in neoplasms of the pancreas are beginning to be integrated with tumor morphology and patient prognosis, and a new “molecular classification” of pancreatic neoplasia is slowly emerging [5]. Acquired genetic mutations may represent new targets for the development of sensitive screening tests for early diagnosis of pancreatic cancer, particularly before it becomes invasive [6,7].

Neural precursor cell expressed developmentally downregulated protein 9 (NEDD9) was initially identified by its developmentally regulated expression pattern in early embryonic, but not adult, mouse brain [8]. Overexpression of NEDD9 protein has now been strongly linked to poor prognosis in various types of cancers, as well as resistance to first-line chemotherapeutics [9,10,11,12,13,14]. There are few articles in the literature about the association of NEDD9 and PC [15,16,17]. It was shown that higher NEDD9 levels were significantly correlated with clinical staging, lymph node metastasis and histological differentiation and patients with a higher NEDD9 expression had a significantly shorter survival time than those patients with lower NEDD9 expression. NEDD9 could serve as an independent factor of poor prognosis in PC patients [15]. MicroRNA-145 suppresses cell proliferation, invasion and migration in PC cells by targeting NEDD9 [16] and baicalein, a flavone ingredient of *Scutellaria baicalensis* Georgi inhibits PC cell proliferation and invasion via suppression of NEDD9 expression and its downstream Akt and extracellular regulated kinase (ERK) signaling pathways [17]. The aim of this study was to investigate the expression and prognostic significance of NEDD9 in PC patients.

## 2. Materials and Methods

### 2.1. Characteristics of the Patients and the Disease

The serum samples of the 32 pancreatic cancer patients with histologically confirmed diseases who were referred to Istanbul University Institute of Oncology and Bakirkoy Dr Sadi Konuk Training and Research Hospital from February 2011 to October 2014 were obtained. The patients had not received chemotherapy and/or radiotherapy over the last 6 months. The stage of the disease was determined according to the American Joint Committee on Cancer (AJCC) and International Union against Cancer (UICC) staging systems. Prior to onset of the treatment, the patients had been processed through a detailed assessment including clinical history, physical examination, and a series of blood tests, such as tumor markers, lactate dehydrogenase, and complete blood count. Investigations were carried out following the rules of the Declaration of Helsinki of 1975, which was revised in 2013. Institutional review board of Istanbul University Institute of Oncology in 2014 with the number 299 was obtained before the study. 

During a median follow-up of 16.5 weeks (range: 1–187 weeks) while 10 patients (31%), experienced disease progression, 21 of the remaining patients (66%) died. Median progression free survival (PFS) and overall survival (OS) of the whole group were 12.0 ± 2.4 weeks (95% CI = 7–17 weeks) and 27.0 ± 7.3 weeks (95% CI = 13–41 weeks), respectively. While 6-months OS rates were 51.1% (95% CI = 33.3–68.9). There were liver metastasis in 16 (50%), abdominal implants in 3 patients, abdominal lymphadenopathy (LAP) in 3, lung in 2, and surrenal in 1 patient. Progression sites were liver in 6 patients, locally in 4 patients, and abdominal LAP in 1 patient.

### 2.2. Statistical Analysis

SPSS for Windows version 21.0 (SPSS Inc., Chicago, IL, USA) was employed for data analysis. Continuous variables were categorized using median values as cut-off point. Relationships and comparisons of several clinical/laboratory variables were evaluated via nonparametric tests. Mann–Whitney U-test was used to assess the serum levels between the subgroups. Overall survival was calculated from the date of first admission to the clinics to disease-related death or date of last contact with the patient or any family member. Progression-free survival was calculated from the date of admission to the date of first radiologic progression with/without elevated serum tumor marker. The Kaplan–Meier method was used for the estimation of survival distribution and differences in PFS and OS were assessed by the log-rank statistics. All statistical tests were carried out two-sided and a *p*-value ≤ 0.05 was considered statistically significant.

## 3. Results

In a three years period, 32 patients with a pathologically confirmed diagnosis of PC were enrolled in this study. The baseline histopathological characteristics and the demographic characteristics of the patients are listed in Table 1. The median age at diagnosis was 61 years, range 38 to 84 years; the majority of the patients in the group were men (n = 20, 62.5%). The tumor was located in the head of pancreas in 21 (65.6%) patients. Forty-one percent of 17 metastatic patients who received palliative CTx were CTx-responsive. Surgery was performed in six (18.7%) patients; three (9%) patients underwent pancreaticoduodenectomy and three (9%) patients had palliative surgery. Serum levels of laboratory parameters in patients were shown in Table 2.

**Figure 1:**
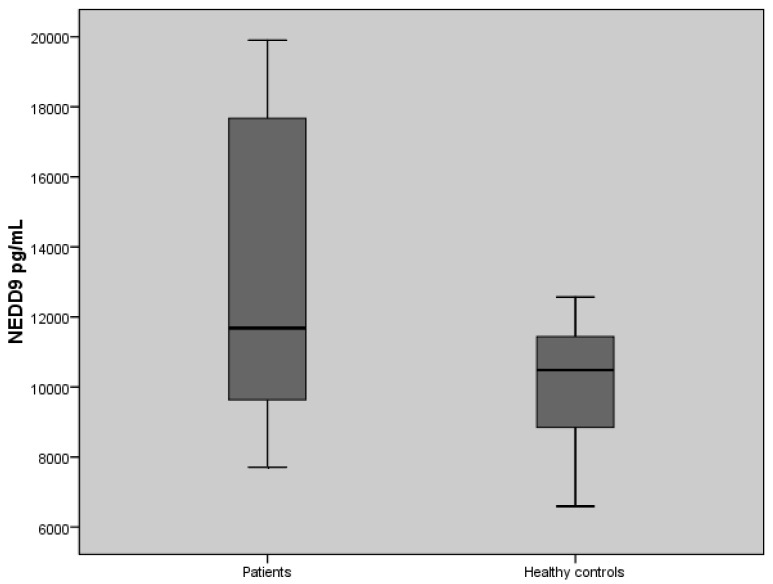
The values of serum neural precursor cell expressed developmentally downregulated protein 9 (NEDD9) assays in pancreatic cancer patients and controls (*p* = 0.03).

The levels of serum NEDD9 assays in patients with PA and healthy controls are shown in Table 3. The baseline serum NEDD9 levels were significantly higher in patients with PA than in the control group (*p* = 0.03) (Figure 1).

**Figure 2:**
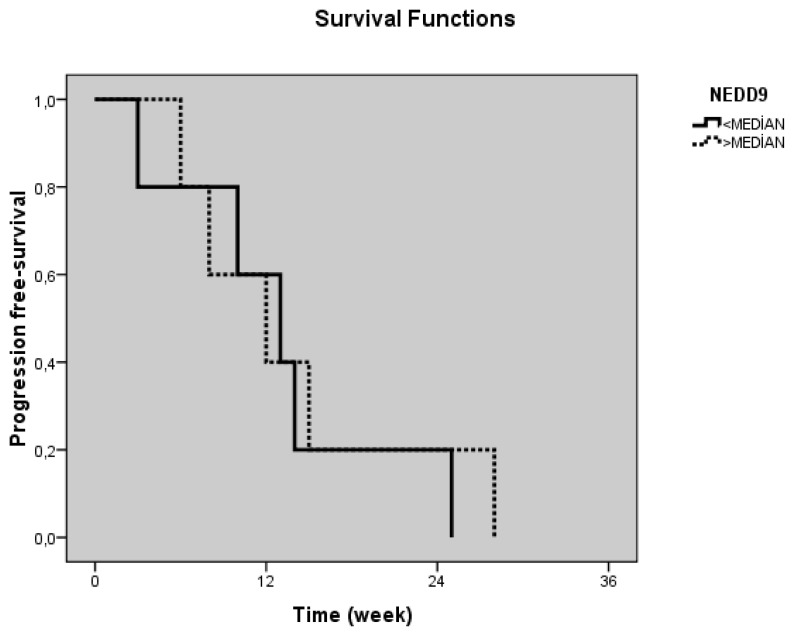
Progression-free survival curves in pancreatic cancer patients according to serum NEDD9 levels (*p* = 0.71).

**Figure 3:**
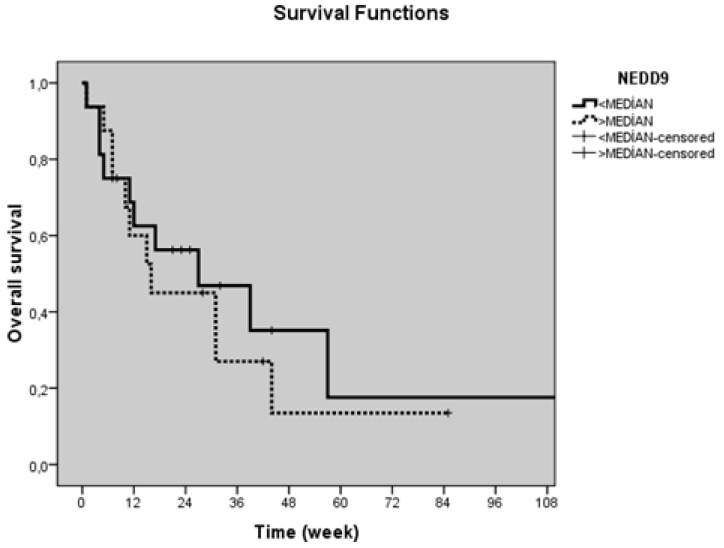
Overall survival curves in pancreatic cancer patients according to serum NEDD9 levels (*p* = 0.58).

Table 4 shows the correlation between the serum levels of NEDD9 and clinicopathological factors. Median OS of the whole group were 27 ± 7.3 weeks. Alcohol intake, performance status, LDH levels were found to be significant prognostic factors (*p* = 0.006, *p* < 0.001, and *p* < 0.001, respectively) (Table 5). However, serum NEDD9 levels had no significantly effect on PFS and OS (*p* = 0.71 and *p* = 0.58, respectively) (Table 5 and Table 6 and Figure 2 and Figure 3).

## 4. Discussion

Pancreatic cancer (PC) is a highly lethal malignancy. Extensive research is being conducted to identify novel diagnostic, predictive, and prognostic biomarkers for PC [18]. NEDD9 supports oncogenic signaling in a number of solid and hematologic tumors. Aurora A kinase (AURKA) is overexpressed in 96% of human cancers and is considered an independent marker of poor prognosis. NEDD9 depletion destabilizes AURKA and heightens the efficacy of Aurora A inhibitors and it has implications for treatment of metastatic solid tumors [19]. NEDD9 has been found to be diagnostic, prognostic, and predictive in different kinds of tumor types [12,20,21,22].

Little is known about the role of NEDD9 in PC but available data suggest elevated NEDD9 levels have been found in PC patients and it can have a prognostic role [15,16,17,18,20]. High levels of expression of NEDD9 were significantly correlated with clinical staging, lymph node metastasis, and histological differentiation in PC patients [15]. NEDD9 expression was not statistically correlated with tumor stage and grade, gender, or patient survival in another study done by Radulović et al. [18].

Novel therapeutic agents are being investigated in PC. Not only the tumor cells but also the stroma is very important in PC. Immunotherapeutic options are limited and we are still using various kinds of chemotherapies. MicroRNA-145 suppresses cell proliferation, invasion and migration in PC cells by targeting NEDD9 [16]. NEDD9 increases the invasiveness of solid tumors such as gastric cancer, ovarian cancer, and glioblastoma [14,20,23]. There is growing evidence that NEDD9 is itself nononcogenic but changes in expression of NEDD9 (most commonly elevation of expression) are common features of tumors, and directly impact tumor aggressiveness, metastasis, and response to at least some targeted agents inhibiting NEDD9-interacting proteins. These data strongly support the relevance of further development of NEDD9 as a biomarker for therapeutic resistance [24].

## 5. Conclusions

According to these data, in our study, we found out that NEDD9 can be used as a diagnostic marker for patients with PC but it has no prognostic role. The limitation of our study is the small sample size.

## Figures and Tables

**Table 1:** Characteristics of the patients and disease.

Variables	*n*
No. of patients	32
Age (years): Median (range)	61 (38–84)
Gender: Male/Female	20/12
Smoking ^a^: Yes/No	17/12
Alcohol intake ^a^: Yes/No	4/26
Comorbidity ^a^: Yes/no	20/10
Weight loss ^a^: Yes/no	16/6
Jaundice: Yes/no	7/25
Performans status ^a^: 0/1/2/3	6/13/6/6
Surgery Type ^aa^: Whipple surgery/palliative surgery	3/3
Tumor size ^a^: <Small (<40 mm)/≥large (≥40 mm)	14/13
Site of lesion: Head/Corpus-tail	21/11
Response to chemotheraphy: Yes (PR + SD)/no(PD)/unknown	7/7/3
Metastasis (*p*): Yes/no	18/14

^a^ Patients with unknown data concerning the variables are not included in the analysis. ^aa^ In 14 patients with nonmetastatic. PR: partial response; SD: stable disease; PD: progressive disease

**Table 2:** Serum levels of laboratory parameters in patients.

Variables	Values of n (%)
WBC ^a^	
High (>10,000)/normal (<10,000)	5/25
Hemoglobin ^a^	
Low (<12)/normal (>12)	11/19
PLT ^a^	
Low (<150,000)/normal (>150,000)	4/26
Lactate dehydrogenase ^a^	
High (>450 IU/mL)/normal (<450 IU/mL	3/23
Albumin ^a^	
Low (<4 g/dL)/normal (>4 g/dL)	20/9
Carcinoembryonic antigen ^a^	
High (>5 ng/mL)/normal (<5 ng/mL)	12/13
CA19.9 ^a^	
High (>38 U/mL)/normal (<38 U/mL)	24/5

^a^ Patients with unknown data concerning the variables are not included in the analysis. WBC: white blood cell; PLT: platelet; IU: international unit; CA: carbohydrate antigen.

**Table 3:** The values of serum marker levels in pancreatic cancer patients and healthy controls.

	Patients (*n* = 32)		Controls (*n* = 20)		
Marker	Median	Range	Median	Range	*p*
NEDD9 (pg/mL)	1168.30	770.70–7523.11	1048.21	659.24–1256.54	0.03 *

* *p* ≤ 0.05.

**Table 4:** Comparisons of serum marker levels according to various clinical/laboratory parameters.

Variables	Values of *n*	NEDD9 (pg/mL) Median (Range)
Age patients (*p*)		0.89
Young (<60)	13	1169.93 (880.57–3936.74)
Older (>60)	19	1166.67 (770.7–7523.11)
Gender (*p*)		0.64
Male	20	1180.56 (770.70–7523.11)
Female	12	1090.69 (904.46–5525.55)
Smoking (*p*)		0.79
Yes	17	1130.72 (770.70–7523.11)
No	12	1168.30 (904.46–6868.61)
Alcohol intake (*p*)		0.86
Yes	4	1078.87 (880.57–6946.47)
No	26	1148.70 (770.70–7523.11)
Comorbidity (*p*)		0.36
Yes	20	1180.56 (821.66–6946.47)
No	10	1030.31 (770.70–7523.11)
PS (*p*)		0.9
Good (0–1)	19	1169.93 (770.70–7523.11)
Worse (2–4)	12	1148.70 (880.57–6946.47)
Weight loss (*p*)		0.88
Yes	16	1128.27 (770.70–7523.11)
No	6	1365.28 (821.66–6946.47)
Jaundice (*p*)		0.26
Yes	7	1014.71 (880.57–7523.11)
No	25	1191.18 (770.70–6946.47)
Surgery (*p*)		0.85
Yes	6	1443.63 (821.66–1965.69)
No	26	1148.70 (770.70–7523.11)
Localization (*p*)		0.86
Head	21	1169.93 (821.66–7523.11)
Corpus-tail	11	1125.82 (770.70–6868.61)
Tumor size (*p*)		0.7
Small (<40 mm)	14	1235.30 (821.66–7523.11)
Large (≥40 mm)	13	1169.93 (770.70–6868.61)
Metastasis (*p*)		0.43
Yes	18	1084.97 (770.70–6946.47)
No	14	1433.01 (821.66–7523.11)
Liver metastasis (*p*)		0.35
Yes	16	1147.88 (904.46–6946.47)
No	2	966.56 (770.70–5525.55)
Hemoglobin (*p*)		0.95
Low	11	1166.67 (952.23–6868.61)
Normal	19	1169.93 (770.70–7523.11)
WBC (*p*)		0.28
High	5	1736.93 (904.46–6868.61)
Normal	25	1130.72 (770.70–7523.11)
PLT (*p*)		0.04 *
Low	4	1247.55 (770.70–7523.11)
Normal	26	959.40 (821.66–1130.72)
Albumin (*p*)		0.6
Low	20	1247.55 (770.70–7523.11)
Normal	9	1066.99 (918.79–6868.61)
Lactate dehydrogenase (*p*)		0.32
High	3	3936.74 (952.23–6868.61)
Normal	23	1169.93 (821.66–7523.11)
Carcinoembryonic antigen (*p*)		0.74
High	13	1150.33 (770.70–6946.47)
Normal	12	1191.18 (821.66–6868.61)
CA 19.9 (*p*)		0.04 *
High	24	1965.69 (1066.99–6946.47)
Normal	5	1148.70 (770.70–6868.61)
Response to chemotherapy (*p*)		0.85
Yes (PR + SD)	7	1166.67 (918.79–1990.20)
No (PD)	7	1044.12 (821.66–5525.55)

* *p* ≤ 0.05.

**Table 5:** Univariate analyses of serum marker progression-free survival.

Parameters	*N* of Events/Total *N*	Survival (Weeks) Median (±SD)	*p*
All patients	10/32	12 (2.4)	
NEDD9			
<Median	5/16	13.0 (3.3)	0.71
>Median	5/16	12.0 (4.4)	

**Table 6:** Univariate analyses of overall survival.

Parameters	N of Events/Total N	Survival (Weeks) Median (±SD)	6-Month Survival (%) (±SD)	*p*
All patients	21/32	27.0 (7.3)	51.1 (9.1)	
Age patients				
Young (<60)	9/13	31.0 (12.3)	68.4 (13.1)	0.33
Older (>60)	12/19	16.0 (4.1)	39.7 (11.5)	
Gender				
Male	11/20	44.0 (23.5)	52.5 (11.6)	0.21
Female	10/12	17.0 (13.9)	50.0 (14.4)	
Smoking				
Yes	11/17	17.0 (11.5)	46.3 (15.0)	0.35
No	8/12	39.0 (20.3)	57.8 (12.2)	
Alcohol intake				
Yes	3/4	1.0 (NR)	NR	0.006 *
No	16/26	31.0 (9.5)	59.2 (10.0)	
Comorbidity				
Yes	13/20	17.0 (12.2)	48.2 (11.4)	0.53
No	6/10	31.0 (9.6)	68.6 (15.1)	
P				
Good (0–1)	8/19	44.0 (8.6)	71.6 (10.8)	<0.001 *
Worse (2–4)	12/12	7.0 (4.3)	25.0 (12.5)	
Weight loss				
Yes	13/16	11.0 (1.8)	34.7 (12.3)	0.11
No	2/6	39.0 (27.7)	83.3 (15.2)	
Jaundice				
Yes	7/7	11.0 (9.2)	28.6 (17.1)	0.09
No	14/25	31.0 (8.4)	57.8 (10.2)	
Surgery				
Yes	4/6	27.0 (7.4)	51.6 (10.1)	0.78
No	17/26	15.0 (16.7)	50.0 (20.4)	
Localization				
Head	13/21	27.0 (10.0)	53.6 (11.4)	0.2
Corpus-tail	8/11	12.0 (7.8)	45.5 (15.0)	
T size				
Small (<40 mm)	9/14	31.0 (9.3)	64.3 (12.8)	0.28
Large (>40 mm)	9/13	12.0 (1.7)	34.6 (13.8)	
Metastasis				
Yes	12/18	12.0 (6.0)	40.7 (12.3)	0.12
No	9/14	31.0 (11.4)	64.3 (12.8)	
Liver metastasis				
Yes	11/16	12.0 (5.6)	39.1 (13.1)	0.31
No	2/2	17.0 (12.3)	NR	
Hemoglobin				
Low	9/11	15.0 (5.1)	36.4 (14.5)	0.06
Normal	10/19	31.0 (11.6)	65.6 (11.4)	
WBC				
High	3/5	11.0 (3.0)	NR	0.36
Normal	16/25	31.0 (13.4)	56.0 (9.9)	
PLT				
Low	2/4	57.0 (NR)	NR	0.6
Normal	17/26	27.0 (8.0)	51.0 (10.0)	
Albumin				
Low	14/20	17.0 (7.9)	45.7 (11.8)	0.07
Normal	4/9	39.0 (NR)	77.8 (13.9)	
Lactate dehydrogenase				
High	3/3	7.0 (0.0)	NR	<0.001 *
Normal	13/23	39.0 (7.3)	68.3 (9.9)	
Carcinoembryonic antigen				
High	10/13	12.0 (3.2)	37.5 (14.7)	0.29
Normal	8/12	31.0 (5.8)	61.5 (13.5)	
CA 19				
High	17/24	27.0 (7.4)	51.6 (10.6)	0.13
Normal	2/5	NR	NR	
Response to chemotherapy				
Yes (PR + SD)	4/7	31.0 (2.6)	71.4 (17.1)	0.25
No (PD)	5/7	39.0 (2.8)	57.1 (18.7)	
NEDD9				
<Median	10/16	27.0 (14.0)	56.3 (12.4)	0.58
>Median	11/16	16.0 (4.4)	45.0 (13.3)	

* *p* ≤ 0.05.
